# Grafting enhances drought tolerance by regulating stress-responsive gene expression and antioxidant enzyme activities in cucumbers

**DOI:** 10.1186/s12870-022-03791-7

**Published:** 2022-08-20

**Authors:** Said A. Shehata, Hanaa S. Omar, Ahmed G. S. Elfaidy, Shereen S. F. EL-Sayed, Mohamed E. Abuarab, Emad A. Abdeldaym

**Affiliations:** 1 Department of Vegetable Crops, Faculty of Agriculture, Cairo University, PO box 12613, Giza, Egypt; 2 Department of Genetics, Faculty of Agriculture, Cairo University, PO box 12613, Giza, Egypt; 3 Department of Agricultural Engineering, Faculty of Agriculture, Cairo University, PO box 12613, Giza, Egypt

**Keywords:** Deficit irrigation, Grafting, *Cucumis sativus*, Agro-physiological properties, Gene expression, Phytohormones

## Abstract

Water scarcity is a major limiting factor for crop yield production in arid and water-stressed areas worldwide. Cucumber plants have a high transpiration ratio and are vulnerable to drought. Grafting commercial genotypes onto selected strong rootstocks has been useful in mitigating the effects of drought. Therefore, this study aimed to evaluate the possibility of using a novel rootstock plant’s tolerance to water-deficit stress in inducing drought tolerance in cucumbers by activating the stress-response gene expression and the antioxidant system, which improved the cucumber quality and yield under water-deficit conditions. This field experiment was conducted for > 2 years, 2020 and 2021, with five drought stress tolerant genotypes (i.e., rootstock) and drought-sensitive genotype Luerans (i.e., a scion). They were subjected to various deficit irrigation levels for 12 days, and their agro-physiological and molecular responses to water-deficit stress were assessed. The results of the study showed that the agronomical parameters, including the leaf area (LA), leaf water content (LWC), number of leaves, plant height, root dry matter shoot dry matter, rates of leaf appearance and stem elongation, and total yield significantly increased with grafted cucumber plants than with non-grafted cucumber plants (control) under normal and stressful conditions.Similar results were observed in the physiological measurements in terms of antioxidant enzymes, abscisic acid levels, gibberellic acid content, and lower lipid peroxidation (malondialdehyde, MDA). Grafting of Luerans (section) on five rootstocks significantly raised the activity of antioxidant enzymes (catalase and peroxidase), improved the gibberellic acid and proline accumulation, and reduced the content of lipid peroxidation and abscisic acid. Furthermore, the real-time polymerase chain reaction expression results revealed that transcript levels of the stress-response genes *CsAGO1* and *CsDCLs* increased rapidly and continuously in five rootstock grafting. Concurrently, these findings suggest that grafting with local varieties of novel drought-tolerant rootstock genotypes could improve drought tolerance in drought-sensitive cucumber genotypes.

## Introduction

Water scarcity is a major issue that several societies and the world are dealing with. In the last century, water consumption has increased more than double than the population growth. In particular, an increasing number of regions are chronically droughted [1–3]. Water deficiency stress is the main serious abiotic stress affecting plant growth and has become the preliminary cause of crop productivity and quality declines [2, 4, 5]. Most plants experienced growth rate, leaf water content (LWC), relative water content, chlorophyll content, photosynthetic frequency, stomatal conductance, and transpiration frequency changes due to water-deficit conditions, which controlled inadequate plant growth and yield production [1, 6, 7]. Antioxidant enzymes, i.e., catalase (CAT), peroxidase (POD), and glutathione reductase (GSR), showed lower activity under water-deficit stress, which causes oxidative tension in plant cells, but increases malondialdehyde concentration, i.e., hydrogen peroxide [8].

Cucumber plants have an extraordinary transpiration ratio and are sensitive to stress, which increases reactive oxalate production [9]. Cucumber (*Cucumis sativus* L.) is the most important crop in the Cucurbitaceae family and contains approximately 800 varieties, including watermelon, melon, squash, pumpkin, and guard, as well as some locally cultivated and wild types. Cucurbita species contain highly valuable components, such as tannins, terpenoids, and antioxidants, such as polyphenols, flavonoids, and carotenoids, which are beneficial to human health as well as in the fight against plant diseases [10, 11].

Recently, several techniques have been extensively used to enhance drought tolerance and nutrient uptake. Grafting with rootstock genotypes maintains plants from water deficits, chilling stresses, and oxidative stresses [3, 12]. Grafting is a vegetative propagation technique where two active plant organisms are combined to grow as a solo plant. The grafting method used in the lower part of the plant is called the rootstock. The rootstock is selected for its genomic capability to fight against abiotic stress. The higher portion of the plant is called a scion. The scion is selected for fruit quality and yield improvement. Grafting is now considered a quick and easy alternative to the moderately leisurely breeding policy for growing the ecological-stress acceptance of ripening vegetables. Grafting high-yielding genetic constitutions onto correct rootstocks capable of controlling the effects of water deficits on the shoot and increasing tolerance is a likely way to reduce production problems and increase water use efficacy. Grafting is widely employed to propagate fruit, nuts, and ornamentals. Nowadays, grafting is also performed on cucurbitaceous and solanaceous crops to afford sickness struggles and increase nutrient uptake and tolerance to ecological tensions on plants, such as temperature, drought, salinity, and flooding [13–15]. To date, no reports have verified the effects of novel rootstock grafting on water-deficit stress in cucumber.

Therefore, the current study aimed to evaluate whether novel grafting with rootstock plants tolerant to water-deficit stress may promote deficiency tolerance in cucumbers by activating the stress-responsive gene expression and the antioxidant system; design water-deficit yield production trials and agronomic approaches to enhance crop water production and discover specific characteristics associated with drought acclimatization of cucumber plants; evaluate five drought-tolerant genotypes as rootstocks and a water-deficit-sensitive genotype, Lureans, as a scion at different deficit irrigation levels for about 12 days; and finally, examine the impacts of grafting and its potential mechanisms on physiological parameters and molecular levels under water-deficit stress.

## Materials and methods

### Plant material and growth conditions

The experiment was performed from September 28^th^ to December 23^rd^ during the two successive seasons of 2019 and 2020 in the climate-controlled Greenhouse at the experimental farm of the vegetable department at the Faculty of Agriculture, Cairo University, Giza, Egypt (latitude 30.05 N, and longitude 31.21E, and mean altitude of 70 m above sea level).

The Laurens cucumber cultivar (*C. sativus* cv)—has been used as a scion (Enza Zaden Company, Enkhuizen, Netherlands). The scion was grafted onto six rootstocks: Super Green (squash hybrid, Modesto Seeds company, Modesto, California 95357, U.S.A.), Just (squash hybrid, American Takii seeds, California, U.S.A.), Battle Guard1 (Legenaria siceraria, PI 491352), Battle Guard 2 (Legenaria siceraria, PI 491365), Watermelon (Citrullus lanatus var. Colocynthoides), and Laurens (cucumber genotype, Enza Zaden, Enkhuizen, Netherlands).

The grafting process was carried out in a private nursery in Badr Center, EL-Beheira governorate, Egypt, using the splice grafting method. The grafted plants were transplanted 28 days after grafting and then transplanted in a plastic pot under a mixture of sand and silt at a 1:1 ratio. Furthermore, before transplanting, the pots were sterilized with a 10% solution of sodium hypochlorite and then placed under the sun until completely dry.

### Environmental conditions

The experiment was conducted in a naturally ventilated greenhouse, where the experiment location was described by associating an arid climate with cool winter and humid summer. The subsequent environmental condition variables were documented daily throughout each cultivated season in the greenhouse: the maximum air temperature, minimum air temperature, average air temperature, relative air humidity, and a variety of sunshine hours throughout the season (from September to December). Every year, the total precipitation was negligible (20 mm) (Table 1).

**Table 1:** Monthly environmental condition variables in greenhouse for the two cultivated seasons

Year	Climate parameter	Month		
		**October**	**November**	**December**
2019	T_min_. (^0^C)	19.1	16	10.2
	T_max_ (^0^C)	32.6	28.2	22.4
	T_ave_ (^0^C)	25.5	22.1	16.3
	RH (%)	57.08	54.54	66.31
	WS (m sec ^1^)	2.2	1.9	2.3
	Solar radiation (W m^−2^)	211.2	178.5	140.4
2020	T_min._ (^0^C)	19.7	14.2	11.4
	T_max_ (^0^C)	33	26.6	23.7
	T_ave_ (^0^C)	26.35	20.4	17.55
	RH (%)	57.28	63.52	60.63
	WS (m sec ^1^)	2.7	1.8	2.1
	Solar radiation (W m^−2^)	216	157.16	144.72

### Crop evapotranspiration

The reference evapotranspiration (ETo) relies on the Penman–Monteith equation [16] and is usually suggested by the Food and Agriculture Organization of the United Nations by applying daily environmental condition parameters measured at a lookout with intervals of 500 m within the experimental space. The ETo has been successfully utilized by [16–19]. The ETo calculator computer code (http://www.fao.org/land-water/databases-and-software/eto-calculator/en/) was used with the meteorological data as input:

1 $${ET}_{^\circ }=\frac{0.408\Delta \left({R}_{n}+G\right) + \Upsilon \left(\frac{900}{T +{ 273}\mathrm{U}_{2}\left({e}_{s}-{e}_{a}\right)}\right)}{\Delta + \Upsilon \left(1 +{ 0.34}\mathrm{U}_{2}\right)}$$

where ETo represents the reference evapotranspiration (mm day^−1^), Rn represents net radiation at the crop surface (MJ m^−2^ day^−1^), G represents soil heat flux density (MJ m^−2^ day^−1^), T represents the mean daily air temperature at 2 m height (°C), U_2_ represents wind speed at 2 m height (ms^−1^), and es Andean represents saturated vapor pressure deficit (kPa). The crop’s evapotranspiration remains calculated using the methodology and processes outlined in the FAO Irrigation and Drainage Paper No. 56 [16] and it already represents the daily plant water consumption through the collection of water evaporation from soil and water transpiration from plant leaf stomata and evaporation:

2 $${\mathrm{ET}}_{\mathrm{C}}={ET}_{O} ({K}_{cb}+{K}_{e})$$

where K_cb_ represents the coefficient of the basal crop, K_e_ represents the coefficient of soil evaporation, and ETo represents the daily reference evapotranspiration (mm day^−1^).

### Experimental design and stress treatments

At 7 days after transplanting, the grafted and nongrafted cucumber plants were subjected to the following three irrigation levels: 1.0 ETc (Full irrigation), 0.75 ETc (moderate drought), and 0.50 ETc (severe drought). To assess how drought and rootstocks affected the cucumber yields and vegetative traits, researchers used sex cucumber grafting pairings with two different deficit watering levels (0.75 ETc, 0.50 ETc, and 1.0 ETc) as control treatment). The Laurens cucumber genotype (*C. sativus*) has been used as a scion, among some of the cucumber grafting rootstocks (Enza Zaden, Netherlands). As rootstocks including the Super Green (squash hybrid, Modesto Seeds Company, Modesto, California 95,357, USA), Just (squash hybrid, American Takii seeds, California, USA), Battle Guard 1 (Legenaria siceraria, PI 491,352), Battle Guard 2 (*Legenaria siceraria*, PI 491,365), and Watermelon genotypes (*Citrullus lanatus* var. *Colocynthoides*) have been used, each treatment includes ten replicates.

### Measurements and estimates

#### Plant growth parameters

At 70 days after transplantation, the effects of various treatments on cucumber plant growth were identified by assessing the leaf area (LA) using a Biovis LA meter application, number of leaves, and maximum plant height, respectively. The following formula was used to calculate the LWC using the drying method:

3 $$LWC=\frac{FW-DW}{FW}\mathrm x\;100$$

where FW represents the plant’s fresh weight and DW represents the plant’s dry weight. Root and shoot dry matters were estimated using the following equation:

4 $$Root\;and\;Shoot\;dry\;matter=\frac{Dry\;weight}{Fresh\;weight}\;\mathrm x\;100$$

The rate of leaf appearance (RLA) and the rate of stem elongation (RSE) were measured based on the following method [20]:

5 $$RLA=\frac{Maximum\;leaves-Minimum\;leaves}{t_2-t_1}$$

where t_2_ and t_1_ represent the maximum and minimum leaves at 70 and 38 days, respectively.

6 $$RSE=\frac{{H}_{2}-{H}_{1}}{{t}_{2}-{t}_{1}}$$

where RSE is the root square error and t2 and t1 are the plant heights at 35 and 70 days, respectively. The total yield, fruit diameter, fruit length, fruit fresh weight, fruit dry weight, and fruit dry matter were all measured when the fruits were harvested. A digital refractometer was used to calculate the total soluble solids (TSS) of cucumber fruits (model PR101, Co. Ltd., Tokyo, Japan).

#### Water productivity (WP)

WP was estimated using the following equation [21]:

7 $$WP=\frac{Y}{{ET}_{c}}$$

where WP represents WP (kg ha^−1^), Y represents the economic yield (kg ha^−1^), and ETc indicates the plant water consumption (m^3^ ha^−1^).

#### SPAD index and chlorophyll fluorescence factors

At 70 days after transplantation, the fourth leaf of six cucumber plants was selected randomly from each treatment to measure SPAD (chlorophyll content), maximum photochemical efficiency of PSII (Fv/Fm), and effective PSII quantum yield (PSII) using a chlorophyll fluorometer (OS1p chlorophyll fluorometer, Opti-Sciences Inc, New Hampshire, USA) and SPAD meter (SPAD 502, Minolta Co, Osaka, Japan), respectively. Three SPAD readings were averaged and carried around the cucumber’s third leaf. The efficiency of the photosystem II was calculated using the following formula:

8 $$The\;photosystem\;IIeffiency=\frac{F_V}{F_M}$$

where Fv is the variable chlorophyll fluorescence ratio and F_M_ is the maximum chlorophyll fluorescence ratio. Using a modulated chlorophyll fluorometer, the estimate process was carried out in a dark environment for 30 min.

#### Electrolyte leakage

The measurement of electrolytes that escaped from the leaves was used to define electrolyte leakage, which agrees with the technique defined by [22]. Cucumber leaves were cut and soaked in 0.4-M mannitol (Merck) liquid for 3 h at room temperature. The initial conductivity (C_1_) was measured using a conductivity meter (ECO 401, Adwa, Romania). The socked samples were boiled for 15 min, and the electrical conductivity (C_2_) was measured for the second time after cooling down. The ion leakage percentage was estimated using the method described by [22]:

9 $$Electrolyte\;leakage\;\left(\%\right)=\frac{c_1}{c_2}\;x\;100$$

#### Leaf nutrients content

The fully expended leaves of six cucumber plants were chosen at random and ground in liquid nitrogen before being frozen at 80 °C for chemical analysis. The essential elements in freeze-dried cucumber leaves were determined (N, P, K, Ca, Mg, Fe, Zn, and Na). 0.5 g of cucumber leaves were digested with an acid solution containing sulfuric and perchloric acids. After heating the solution for 10 min at 50 °C, 0.5 mL of perchloric acid was counted and the heating persisted until a pure solution was achieved. The nitrogen content in leaf tissues was determined using the Kjeldahel method, according to AOAC [23]. The chlorostannous molybdophosphoric blue color technique was used to estimate phosphorus (P) in leaf tissues. A flame photometer has been used to determine the potassium (K) content (CORNING M410, Essex, UK). Concentrations of Ca, Mg, Fe, Zn, and Na were determined using anatomic absorption spectrophotometer with air-acetylene fuel (Pye Unicam, model SP-).

#### Malondialdehyde (MDA) concentration

A technique was used to assay the amount of MDA in the plant leaves was defined by Heath [24]. In 5 mL of 1% trichloroacetic acid (TCA), 0.2 g of plant leaves were pulverized. The liquid was centrifuged at 10,000 g for 5 min. The liquid was centrifuged at 10,000 g for 5 min. The supernatant was supplemented with 4 mL of 20% TCA, which included thiobarbituric acid. Then, the liquid was elevated in a water bath at 95 °C for 30 min before being directly chilled on ice. Thereafter, the mixture was centrifuged for 10 min at 10,000 g. A spectrophotometer (Varian Cary 50 UV–Vis, Germany) was used to measure MDA in the mixture at wavelength 532 nm. The MDA content was expressed as nanomoles/gram of fresh weight.

#### Gibberellic acid (GA) and abscisic acid (ABA) content

Fales et al. [25] devised procedures for determining the GA concentration and ABA compounds in cucumber leaves. Cucumber samples were freeze-dried, pulverized, and then mixed with methanol (80% v/v, 15 mL g^−1^) and butylated hydroxytoluene at 4 °C in the dark. Additional information on GA and ABA abstraction, resolution, and quantification can be found elsewhere [26].

#### Activity of antioxidant and non- antioxidant enzymes (proline content)

A 0.5 g of fresh leaf samples was powdered in liquid nitrogen and homologized in 5 ml of potassium phosphate buffer (100 mM, pH 7.0) including 0.5% Triton X-100, 2% (w/v) N-Vinylpyrrolidinone, 5 mM Methylene diaminete traacetic acid disodium salt dried and 1 mM ascorbic acid [1]. Homogenates were then centrifuged at 12,000 for 20 min at 4 °C and the supernatants were used to verify the interest of catalase (CAT, EC1.11.1.6) according to Aebi. As previously explained [27], all spectrophotometric analyses were carried out.

The peroxidase amount was quantified using the method described by Aebi [28], in which the peroxidase enzyme was isolated by freezing (0.5 g) in liquid nitrogen. The trials were crushed and centrifuged at 3930 rpm for 20 min with 10 mL of extraction buffer (50 mM phosphate buffer, pH 7, including 0.5 mM EDTA and 2 percent PVPP (w/v)). The peroxidase action was defined using a spectrophotometric method based on the structure of guaiacol in a l mL effect mixture (450 l 25 mM guaiacol, 450 l 225 mM H_2_O_2_) and 100 l of crude enzymes.

The proline content was measured according to the method of Bates (1973) (23). The 100 mg of plant material was homogenized in 1.5 ml of 3% sulphosalicylic acid and then centrifuged.100 mL of extract was combined with 2 mL of glacial acetic acid and 2 mL of acid ninhydrin (1.25 g ninhydrin warmed in 30 mL of glacial acetic acid and 20 ml of 6 M phosphoric acid until dissolved) for 1 h at 100 C, and the reaction was then put into an ice bath. The reaction mixture was extracted with 1 ml of toluene and the absorbance was determined at 520 nm. The proline content was measured according to a standard curve.

### Plant defense genes expression

To better understand the regulation and molecular mechanisms of rootstock grafting during drought, two stress-responsive genes, Argonaut (*CsAGO1*) and RNA-dependent RNA polymerase (*CsRDR1*), were chosen and their transcript analysis was performed using quantitative real-time PCR. These genes have been shown in *Cucumis sativus* plants to respond to drought. RNA was extracted from two-month-old grafting plants collected during twelve days of water deficit stress. A total of 100 mg of cucumber plants were isolated using the RNeasy® Plant Mini Kit (Qiagen, Hilden, Germany). The RNA concentrations were measured using a Nanodrop ND-100 spectrophotometer.

The Prime-Script First Stand cDNA Synthesis Kit was used to convert RNA to cDNA (thermo kit). The cDNA synthesis reactions were incubated at 37 degrees Celsius for 15 min and at 85 °C for 5 s. The cDNA obtained was used in qRT-PCR experiments. Primers specific to two stress-responsive genes were used for PCR amplification. Primer blast (https://www.ncbi.nlm.nih.gov/tools/primer-blast/) or primer3 (https://primer3.ut.ee/) software was used to design primers specific to drought expressed related genes. The primers were used to identify bands of 100–250 bp in length. The *CsAGO1b* gene primer sequences were 5-ACACCGTGGAAATTGTTAGGC-3 and 3-ACTTGAAGGCAAGGGAGATG-5.

The CsDCL1 gene primer sequences were 5-CGGTTTGAACACGCAGAA-3 and 3-GAGGCAGGAACAGGGTAG. *CsActin* 5-CAACCACAAGGGCTAACAGAG-3 and 3-GAATCCAGCACGATACCAGT were the housekeeping gene sequences (5). The Thermal Cycler Bio rad Real-Time System II (TaKaRa, Shiga, Japan) and the SYBR kit were applied for real-time PCR. In 96-well plates, the QRT-PCR analysis was done in triplicate. For a total volume of 25 µl, 12.5 µl SYBR, 1 µl of 60 ng cDNA, 5 µL of 2 mol L-1 primer, and 6.5 µl of DNase free nuclease water were added. The real-time system's thermal profile was comprised one step at 95 °C for 30 s, then 40 cycles at 95 °C for 5 s, and 60 °C for 30 s. As an internal standard, the actin gene was employed (housekeeping gene).

### Statistical analyses

A factorial experiment was designed using the randomized complete design with ten replicates. Analysis of variance and mean comparison were conducted using the MSTAT C v.2.1 programs, followed by Duncan multiple range tests (*P* ≤ 0.05) to determine significant differences. Pearson’s correlation coefficient (r) was performed to determine the relationship between all studied traits. Furthermore, expression analyses were successfully recognized using the average cycle threshold (CT). The CT value determined using the average CT value of genes from that of the housekeeping gene (actin) was estimated from the triplicate experiment accompanied by each gene. Finally, expression analyses were calculated using the delta-delta CT.

## Results

### *Response of *ETc* to climate, crop, and soil parameters*

**Fig. 1:**
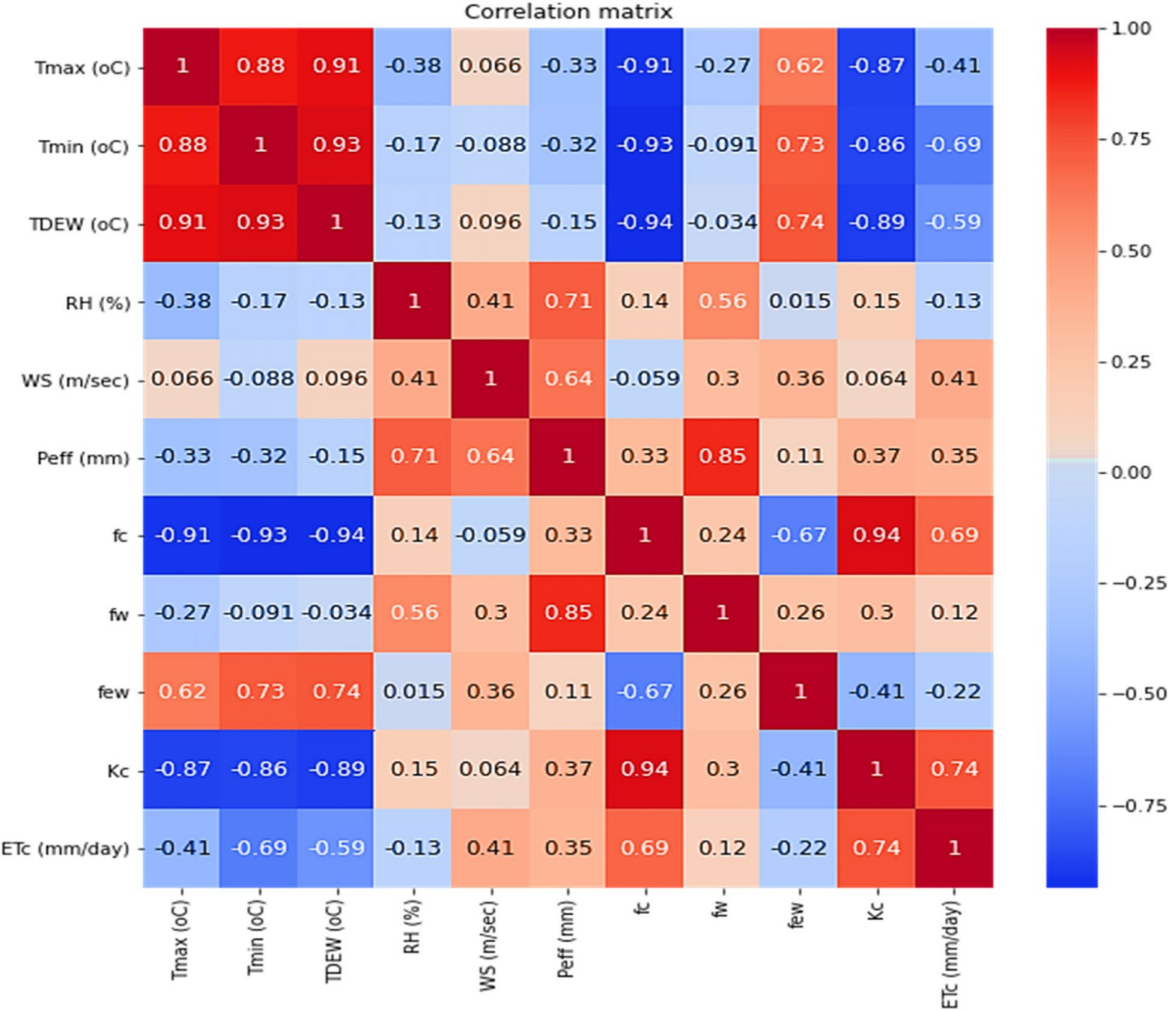
Correlation matrix between maximum temperature, minimum temperature, wind speed, relative humidity, and crop evapotranspiration

Correlation coefficients between crop evapotranspiration [ETc] and climatic parameters (effective precipitation [P_eff_], the average fraction of soil surface covered by vegetation [fc], wind speed [WS], fraction of the surface wetted [f_w_], crop coefficient [K_C_], and ETc) are positive (Fig. 1). The highest correlation coefficient was 0.74 between ETc and K_C_ and 0.69 between ETc and fc, whereas the fraction of the wetted surface had very low positive correlations with ETc, at 0.12. Because the growing period is during winter and Egypt is located in an arid zone, the effective precipitation had a low correlation with ETc, at 0.35. The minimum temperature (Tmin) had the highest negative correlation coefficient of − 0.69 with ETc, and the dew point temperature (T_dew_) and the maximum temperature had the second and third highest negative correlation coefficients of − 0.59 and − 0.41 with ETc. From the analysis of correlation coefficients, the crop, followed by soil, and finally climate parameters, had the greatest impact on ETc, with Kc having the highest impact as a crop parameter, followed by fc as a soil parameter, and finally WS and P_eff_ as climate parameters.

### Cucumber yield and WP

**Fig. 2:**
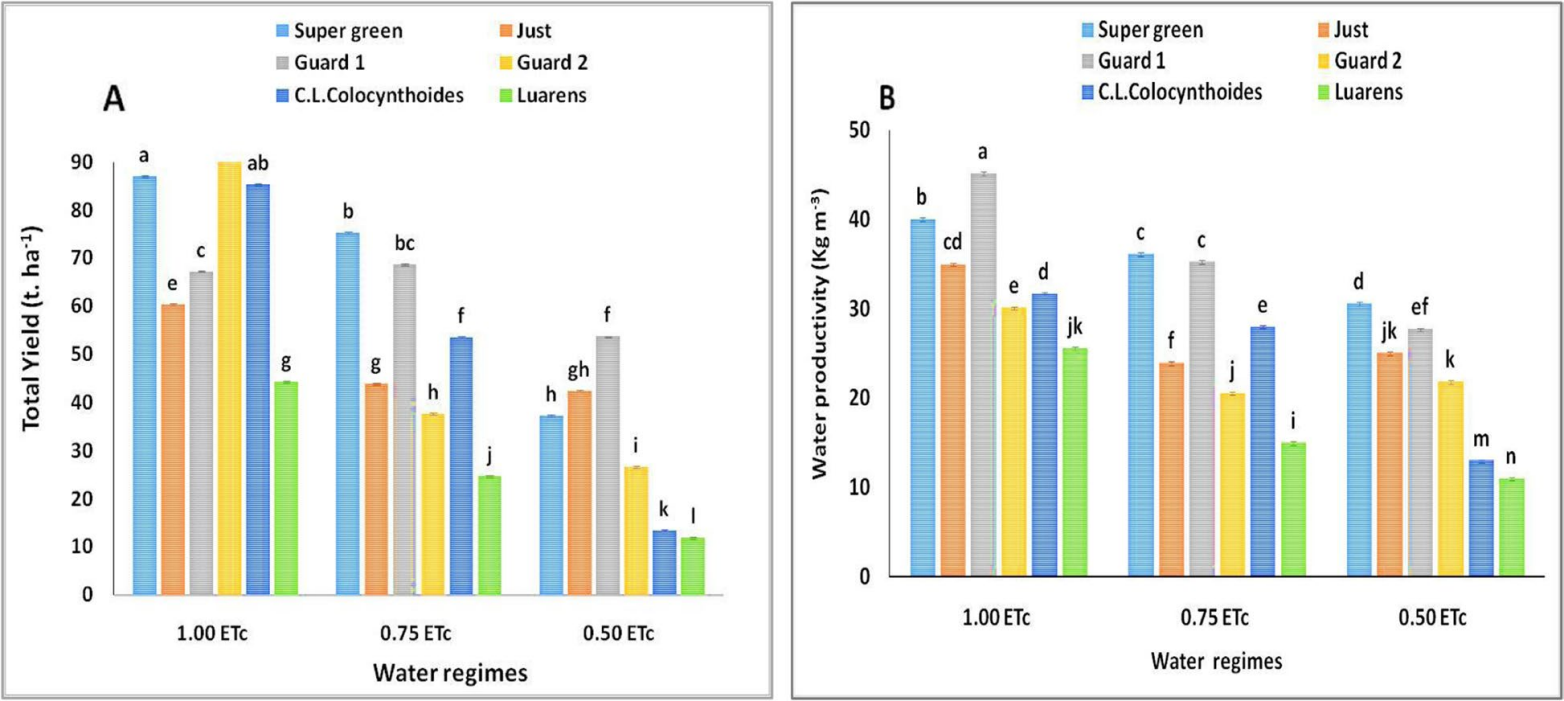
The total yield (**A**) and water productivity (**B**) for different stress levels and plant rootstocks. Different letters indicate significant differences between treatments (Duncan’s multiple range test *p* < 0.05)

Results of the rootstock-grafted plants displayed that the yields significantly varied among plants (Fig. 2), where Super Green and C.L. Colocynthoides rootstock-grafted plants overextended had the highest yield under 1.0 ETc and Super Green and Gurad 1 overextended had significantly highest yield under drought stress (0.75 ETc) and (0.50 ETc) respectively, whereas Laurens registered the lowest yield for both non-drought and drought stress conditions. Furthermore, the results exhibited significant differences among drought stress levels in yield at 1.0 ETc and 0.50 ETc, whereas the yield for both levels of 0.75 ETc overextended other levels in the two seasons had the highest and lowest yields, respectively. Furthermore, the 50% drought stress levels exhibited no significant differences in yield during the two seasons.

### Plant growth characteristics and yield components

The results revealed that grafted plants were more vigorous than nongrafted plants, as indicated by their LA, LWC, number of leaves, plant height, root dry matter shoot dry matter, RLA and RSE, total yield, fruit diameter, fruit fresh weight, fruit length, fruit dry weight, fruit dry matter, and TSS under normal and stressed conditions (Tables 2 and 3).

**Table 2:** Effect of interaction of water regimes and rootstocks on vegetative growth. Different letters indicate significant differences between treatments (Duncan’s multiple range test at *p* < 0.05) and ± value indicates to stander error

Root stock	Treatment	Plant height (cm)	Shoot DM (g)	Root DM (g)	NO. of leaves	LA (cm^2^)	LWC (%)	R LA (Leaf tiller^−^.day^−1^)	RSE (Cm tiller^−^.day^−1^)
**Super green**	**1.00 ETc**	145.3 ± 0.1 a-d	55.95 ± 4.80 a-c	52.97 ± 7.09 a-d	30.87 ± 3.06 a-d	20.87 ± 0.36 ab	84.34 ± 0.27 a-d	0.2767 ± 0.09 b-e	1.567 ± 0.07 a-c
	**0.75 ETc**	140.7 ± 7.7 a-d	46.36 ± 14.26 a-d	48.26 ± 7.05 a-d	29.33 ± 0.58 b-e	19.50 ± 0.67 a-e	84.13 ± 0.74 a-d	0.260 ± 0.01 d-f	1.53 ± 0.32 b-d
	**0.50 ETc**	138 ± 12.0 a-e	35.41 ± 7.25 cd	32.76 ± 6.40 ef	28.63 ± 1.15 b-f	15.07 ± 2.56 f	83.16 ± 0.81 de	0.2233 ± 0.04 ef	1.49 ± 0.19 cd
**Just**	**1.00 ETc**	160 ± 50.7 a-c	58.47 ± 15.03 a-c	53.53 ± 0.11 a-d	35.00 ± 2.52 a	20.54 ± 4.18 a-c	84.52 ± 0.29 ab	0.333 ± 0.05 a-d	1.537 ± 0.39 b-d
	**0.75 ETc**	149.3 ± 24.6 a-d	56.54 ± 17.81 a-c	51.77 ± 12.74 a-d	32.20 ± 8.08 a-c	19.81 ± 1.98 a-d	83.44 ± 0.18 b-e	0.2667 ± 0.08 c-f	1.537 ± 0.36 b-d
	**0.50 ETc**	126.3 ± 30.81b-e	34.67 ± 16.12 cd	41.09 ± 5.49 d-f	25.33 ± 2.0 e–g	17.31 ± 0.67 a-f	83.24 ± 1.00 c-e	0.2300 ± 0.01 ef	1.36 ± 0.16 c-f
**Guard 1**	**1.00 ETc**	156 ± 36.1 a-c	63.32 ± 18.85 ab	59.59 ± 1.28 a	33.34 ± 2.7 ab	17.08 ± 0.01 a-f	85.28 ± 0.91 a	0.3633 ± 0.03 a	1.89 ± 0.32 a
	**0.75 ETc**	145 ± 21.6 a-d	46.78 ± 21.33 a-d	55.13 ± 20.90 a-c	27.33 ± 3.6 c-f	15.33 ± 0.74 ef	85.06 ± 0.90 a	0.2400 ± 0.02 ef	1.56 ± 0.10 a-c
	**0.50 ETc**	125.7 ± 0.1 b-e	46.15 ± 0.1 a-d	43.83 ± 0.1 b-f	25.74 ± 1.73 d-g	14.06 ± 0.64 f	83.76 ± 0.11 b-e	0.2233 ± 0.10 ef	1.19 ± 0.07 d-g
**Guard 2**	**1.00 ETc**	145 ± 57.9 a-d	59.28 ± 19.40 a-c	52.02 ± 4.37 a-d	29.32 ± 2.98 b-e	21.16 ± 4.31 ab	85.20 ± 0.31 a	0.3267 ± 0.02 a-d	1.44 ± 0.20 c-e
	**0.75 ETc**	142.7 ± 9.7 a-d	39.02 ± 29.77 b-d	56.11 ± 14.17 ab	28.89 ± 0.19 b-f	15.70 ± 2.83 d-f	84.19 ± 0.87 a-d	0.2833 ± 0.05 b-e	1.31 ± 0.03 c-f
	**0.50 ETc**	139 ± 14.7a-d	35.77 ± 23.93 cd	41.53 ± 6.72 d-f	24.00 ± 2.84 fg	13.99 ± 5.37 f	83.44 ± 0.19 b-e	0.2133 ± 0.02 ef	1.02 ± 0.11 fg
**C.L. Colocynthoides**	**1.00 ETc**	175.7 ± 5.2 a	56.51 ± 9.95 a-c	51.98 ± 3.72 a-d	31.67 ± 1.73 a-c	20.89 ± 1.96 ab	84.39 ± 1.64 a-c	0.3500 ± 0.05 ab	1.89 ± 0.15 a
	**0.75 ETc**	166 ± 8.4 ab	37.71 ± 22.20 b-d	49.15 ± 1.02 a-d	30.12 ± 1.75 a-e	16.84 ± 0.47 b-f	83.64 ± 1.06 b-e	0.3367 ± 0.06 c-f	1.88 ± 0.32 ab
	**0.50 ETc**	114.3 ± 14.98 de	36.60 ± 7.22 cd	45.39 ± 4.07 b-e	24.08 ± 3.77 fg	15.33 ± 3.05 c-f	83.39 ± 1.59 b-e	0.2500 ± 0.03 ef	1.12 ± 0.18 e–g
**Luarens**	**1.00 ETc**	152.7 ± 17.6 a-d	67.28 ± 25.28 a	59.69 ± 9.19 a	30.87 ± 0.84 a-d	21.45 ± 2.43 a	84.52 ± 0.23 ab	0.2867 ± 0.01 b-e	1.35 ± 0.06 c-f
	**0.75 ETc**	122.7 ± 12.7 c-e	34.75 ± 5.50 cd	41.66 ± 9.66 c-f	26.08 ± 2.00 d-g	11.06 ± 3.61 g	83.36 ± 0.63 b-e	0.2400 ± 0.03 ef	1.26 ± 0.15 c-g
	**0.50 ETc**	86.67 ± 22.4 f	27.85 ± 0.1 d	21.61 ± 0.1 g	18.67 ± 5.02 h	8.96 ± 1.17 h	72.68 ± 0.25 f	0.1533 ± 0.01 g	0.63 ± 0.14 h

**Table 3:** Effect of interaction of water regimes and rootstocks on total yield and its components. Different letters indicate significant differences between treatments (Duncan’s multiple range test at *p* < 0.05) and ± value indicates to stander error

Root stock	Treatment	Total yield (Kg plant ^1^)	fruit diameter (mm)	fruit dry matter (%)	fruit dry weight (g)	fruit fresh weight (g)	fruit length (cm)	TSS (Brix^O^)
**Super green**	**1.00 ETc**	1960 ± 52.08 a	2.77 ± 0.02 a-c	6.093 ± 0.17 a-c	2.333 ± 0.05 a-d	51.0 ± 0.1 a	12.33 ± 0.84 ab	5.270 ± 0.08 ef
	**0.75 ETc**	1696 ± 0.21b	2.697 ± 0.01 a-c	5.653 ± 0.46 a-e	2.267 ± 0.57 a-e	39.78 ± 12.70 a-d	10.11 ± 0.50 c-e	5.463 ± 0.19 cd
	**0.50 ETc**	839.2 ± 60.17 f–h	2.583 ± 0.51 bc	5.547 ± 0.19 a-e	2.120 ± 0.06 a-e	35.06 ± 1.53 a-d	9.117 ± 0.17 ef	5.653 ± 0.12 b
**Just**	**1.00 ETc**	1357 ± 34.36 cd	2.953 ± 0.62 ab	6.227 ± 0.14 ab	3.003 ± 1.11 a	53.67 ± 17.76 a	12.83 ± 1.39 a	5.270 ± 0.07 ef
	**0.75 ETc**	986.7 ± 85.57 e–g	2.683 ± 0.05 a-c	5.653 ± 0.42 a-e	2.210 ± 0.69 a-e	42.03 ± 11.06 a-c	12.33 ± 1.04 ab	5.500 ± 0.12 b-d
	**0.50 ETc**	903.3 ± 44.80 fg	2.500 ± 0.13 c	5.253 ± 0.61 c-f	1.443 ± 0.68 b-e	24.44 ± 14.57 b-d	9.917 ± 1.50 c-e	5.600 ± 0.07 bc
**Guard 1**	**1.00 ETc**	1457 ± 71.07 b-d	2.733 ± 0.06 a-c	5.503 ± 0.29 a-f	2.197 ± 1.13 a-e	41.00 ± 21.20 a-c	11.40 ± 0.43 a-c	5.070 ± 0.07 gh
	**0.75 ETc**	1203 ± 26.76 de	2.600 ± 0.28 bc	5.347 ± 0.36 b-f	1.833 ± 0.54 a-e	34.64 ± 9.54 a-d	10.59 ± 0.75 b-e	5.500 ± 0.10 b-d
	**0.50 ETc**	832.2 ± 60.17 f–h	2.393 ± 0.01 c	5.227 ± 0.24 c-f	1.743 ± 0.63 a-e	30.07 ± 7.80 a-d	10.00 ± 0.29 c-e	5.533 ± 0.01 b-d
**Guard 2**	**1.00 ETc**	1546 ± 30.51 bd	2.800 ± 0.32 a-c	5.840 ± 0.75 a-d	2.050 ± 0.76 a-e	35.50 ± 16.83 a-d	11.09 ± 1.06 a-d	5.370 ± 0.05 de
	**0.75 ETc**	849.2 ± 52.94 e–h	2.740 ± 0.10 a-c	5.640 ± 0.21 a-e	2.167 ± 0.58 a-e	33.28 ± 18.12 a-d	10.00 ± 0.1 c-e	5.500 ± 0.10 b-d
	**0.50 ETc**	600 ± 69.92 hi	2.683 ± 0.02 a-c	4.760 ± 0.56 ef	1.137 ± 1.90 c-e	23.14 ± 31.21 b-d	8.00 ± 2.62 fg	6.133 ± 0.01 a
**C.L. Colocynthoides**	**1.00 ETc**	1740 ± 30.41 ab	3.057 ± 0.04 a	6.360 ± 0.90 a	2.673 ± 0.40 ab	52.67 ± 8.21 a	11.01 ± 0.50 a-d	5.170 ± 0.12 fg
	**0.75 ETc**	1207 ± 70.87 de	2.717 ± 0.25 a-c	5.240 ± 0.98 c-f	2.520 ± 0.23 a-c	46.33 ± 2.70 ab	9.517 ± 1.06 d-f	5.400 ± 0.07 de
	**0.50 ETc**	356.7 ± 24.01 i	2.400 ± 0.01 c	5.093 ± 0.16 d-f	1.103 ± 0.43 de	19.00 ± 3.61 cd	9.180 ± 1.35 ef	6.033 ± 0.08 a
**Luarens**	**1.00 ETc**	1316 ± 79.17 cd	3.033 ± 0.28 a	5.693 ± 1.30 a-e	2.323 ± 0.80 a-d	41.67 ± 14.12 a-c	11.50 ± 1.42 a-c	5.000 ± 0.20 h
	**0.75 ETc**	712.2 ± 21.20 gh	2.570 ± 0.25 bc	4.920 ± 0.46 d-f	1.570 ± 1.38 b-e	33.04 ± 13.84 a-d	9.167 ± 1.00 ef	5.183 ± 0.06 fg
	**0.50 ETc**	330 ± 113.17 i	1.667 ± 0.18 d	4.273 ± 0.10 g	0.713 ± 1.23 f	13.67 ± 20.36 e	5.833 ± 0.17 h	5.400 ± 0.01 de

The influence of grafting on cucumber growth parameters exhibited a positive interconnection between the rootstock and scion. The drought treatments (0.50 and 0.75ETc) significantly reduced all growth parameters of both grafted and nongrafted plants. However, the impact of drought treatments was more pronounced in nongrafted than grafted cucumber plants.

Under normal and stressful conditions, the highest values of growth parameters, including leaf area (LA), leaf water content (LWC), number of leaves, plant height, root dry matter shoot dry matter, leaf appearance (RLA), and rate of stem elongation RSE were observed in grafted plants compared to nongrafted cucumber plants.Compared to severe drought treatment (0.50 ETc), the maximum rate for the previous growth traits was observed when grafted cucumber plants on the rootstock of Just, Battle Guard 1, Battle Guard 2, and Watermelon (Colocynthoides) under the full irrigation treatment, followed by the moderate treatment (0.75ETc).

Similar trends were observed in the total yield and its components. Grafted cucumber plants significantly enhanced the total yield, fruit diameter, fruit dry matter, fruit dry weight, fruit fresh weight, and total soluble solids (TSS) compared to nongrafted under normal and stressful conditions. (Table 3). Under full irrigation conditions, the maximum values for the total yield and its components, except fruit TSS, were recorded when grafted on the super green, followed by C.L. Colocynthoides, Guard 2, Guard 1, and Just compared to nongrafted. The TSS value gradually increased with reducing water quantity. The highest TSS values were recorded in the fruit of cucumber plants that were grown under severe drought conditions (0.50 ETc) in comparison to full and moderate irrigation treatments*.*

### Physiological traits

#### Chlorophyll content, photosystem II efficiency, and chlorophyll fluorescence

**Fig. 3:**
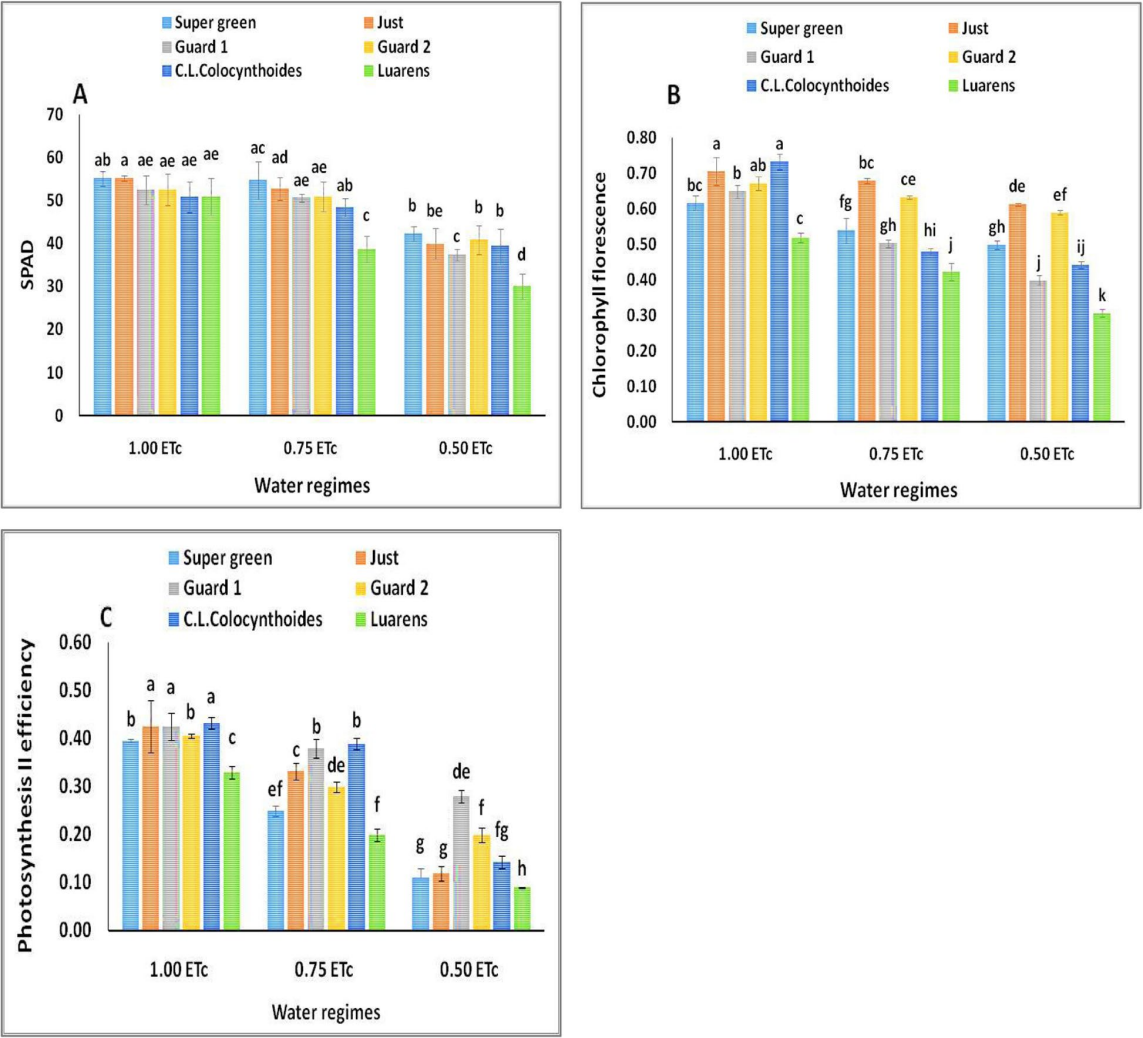
Effect of interaction between water regimes and rootstocks on (**A**) SPAD, (**B**) chlorophyll florescence (Fv/Fm), and (**C**) photosynthesis II efficiency. Different letters indicate significant differences between treatments (Duncan’s multiple range test *p* < 0.05)

In the current study, leaf chlorophyll content (SPAD), Photosystem II efficiency (PSII), and chlorophyll fluorescence (Fv/Fm) were affected by the used rootstocks and water levels (Fig. 3). These results showed higher leaf chlorophyll content (SPAD, Fig. 3A), chlorophyll fluorescence (Fig. 3B) and PSII efficiency (Fig. 3C) content recorded in grafted cucumber than in nongrafted plants under normal and stressful conditions (Table 3). While reduction of the aforementioned parameters was observed clearly in the leaves of nongrafted cucumber plants combined with moderate and severe droughtes (0.50 and 0.75 ETc), which is described as a typical sign of oxidative stress, resulting in photooxidation and chlorophyll degradation of the pigment.

#### Membrane stability and lipid peroxidation (MDA)

**Fig. 4:**
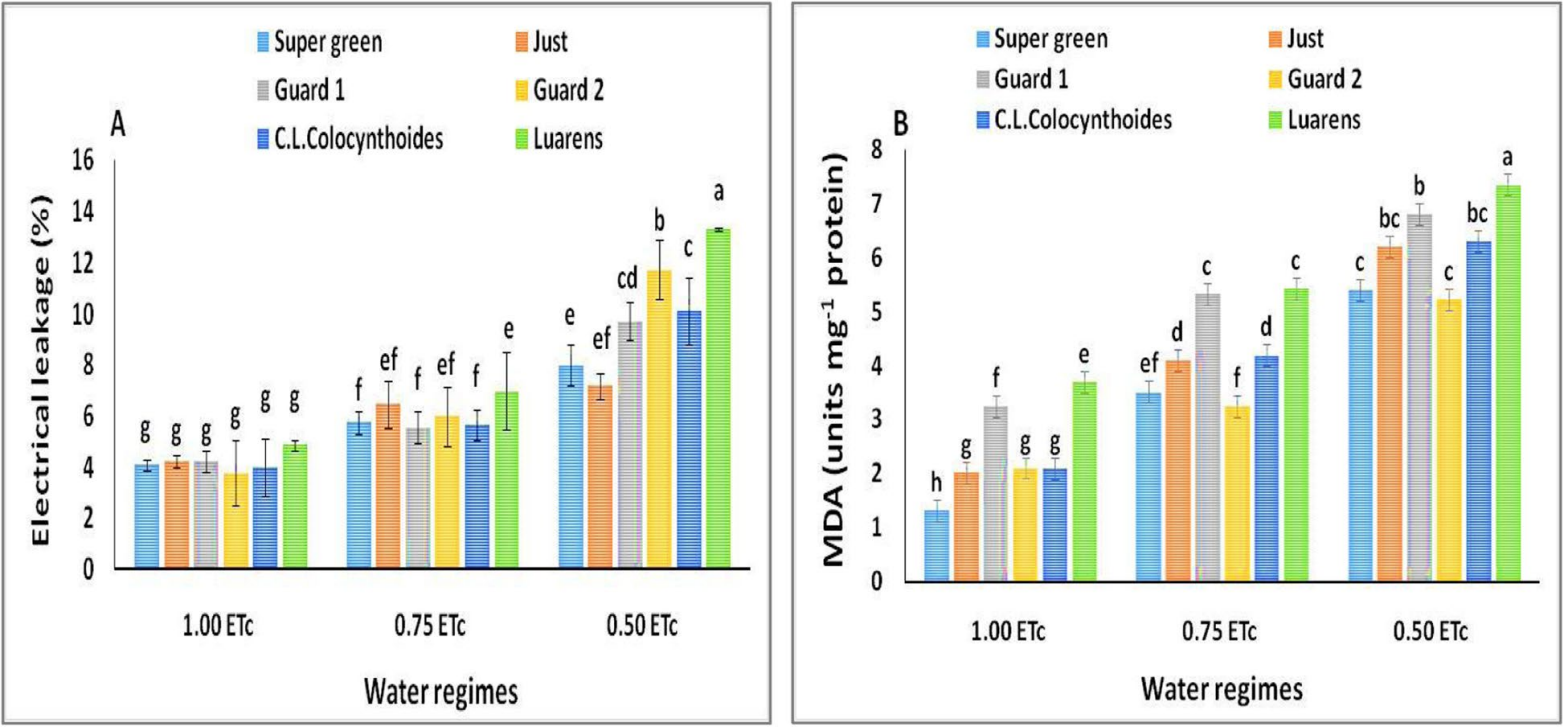
Effect of interaction between water regimes and rootstocks on (**A**) electrical leakage and (**B**) MDA. Different letters indicate significant differences between treatments (Duncan’s multiple range test *p* < 0.05)

Membrane stability, as exhibited by the ratio of electrolyte leakage (EL%), was observed to be significantly affected under normal and stressful conditions (Fig. 4A). Results showed that the EL% was demonstrated to be high in nongrafted plants as compared to the other five grafted plants, particularly under drought conditions (0.50 and 0.75 ETc).

The MDA concentration during 12 days of drought stress, a measure of lipid peroxidation, was estimated. The MDA content significantly increased by reducing the irrigation water levels (Fig. 4B). Under stress conditions, a higher MDA value was noted in nongrafted cucumber plants in comparison to grafted cucumber plants. The lowest MDA contents were observed when grafted cucumber on super green and Guard2 rootstocks combined with moderate and severe water treatments.

#### ABA and GA

**Fig. 5:**
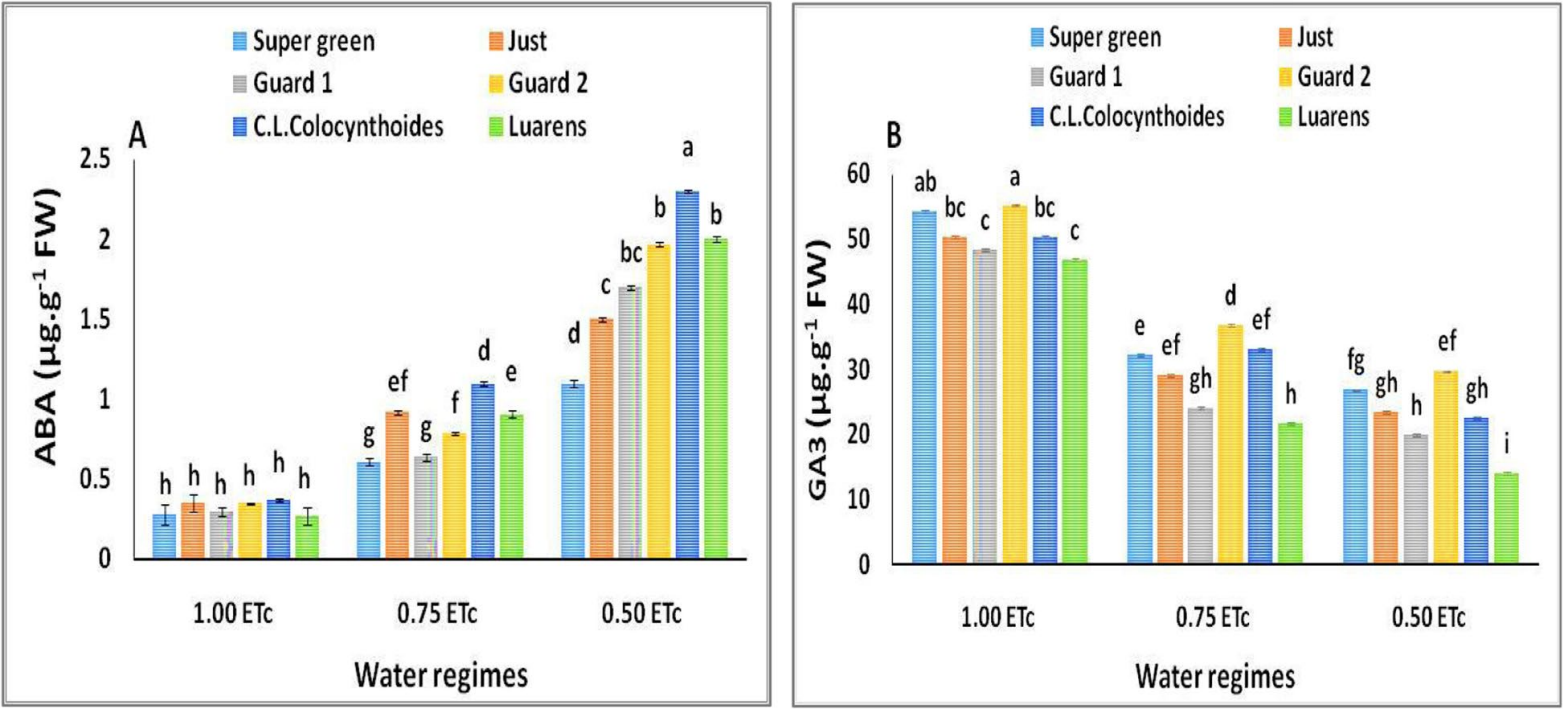
Effect of interaction between water regimes and rootstocks on (**A**) gibberellic acid (GA3) and (**B**) Abscisic acid (ABA).Different letters indicate significant differences between treatments (Duncan’s multiple range test at *p* < 0.05)

The dynamic changes of plant hormones, such as ABA and GA3 levels, were evaluated in the nongrafted and investigated rootstock-grafted genotypes during 12 days of drought treatment (0.50 ETc and 0.75 ETc). Under drought conditions, ABA levels were significantly increased among the five grafted plants, whereas the magnitudes of these increases were different. As the nongrafted plants exhibited a clear decrease in the ABA level (0.50 ETc and 0.75ETc) (Fig. 5A). the results revealed that no differences in ABA content were shown between grafted plants compared to nongrafted under full irrigation treatment (1.0 ETc).

Conversely, for GA3 content, the grafted plants exhibited a highly significant increase in the GA3 concentrations compared to nongrafted plants under normal and stressful conditions (Fig. 5B). Generally, the highest GA3 value was detected in grafted plants combined with full irrigation treatments as compared to all other treatments. Furthermore, significant differences were observed between grafted plants in the GA3 concentration. The highest GA3 content was observed on the cucumber plants grafted on Guard 2 and super green under different irrigation water levels, as compared to all grafted and nongrafted plants. It was also observed that the GA3 content significantly reduced gradually with decreasing the irrigation water levels.

#### CAT, POD and proline content

**Fig. 6:**
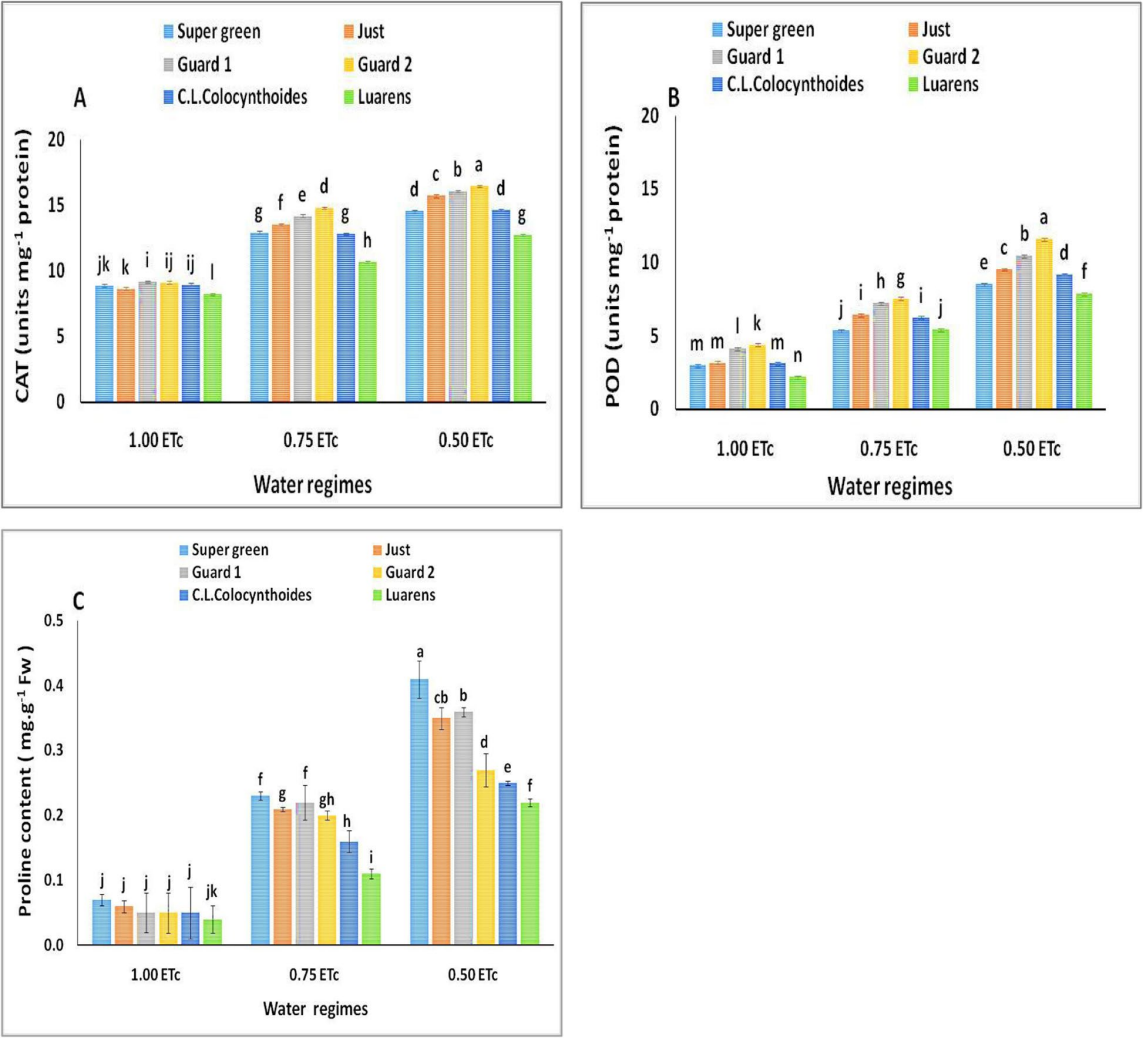
Effect of interaction between water regimes and rootstocks on (**A**) Catalase enzyme activity (CAT) and (**B**) peroxidase enzyme activity (POD), (**C**) proline content. Different letters indicate significant differences between treatments (Duncan’s multiple range test at *p* < 0.05*)*

The antioxidant enzymes were significantly affected by the water shortage and used rootstocks (Fig. 6). During 12 days of drought stress, the dynamic change in CAT was assessed in nongrafted and five rootstock-grafted plants (Fig. 6A). The CAT enzyme activity significantly increased regularly with reducing the irrigation water level. Under drought conditions, grafted plants had higher catalase enzyme activity than nongrafted plants (Fig. 6A). The highest CAT enzyme values were noted in plants grafted on Gurad 2, followed by Guard 1, Just, Super green, and Colocynthoides rootstocks compared to nongrafted cucumber plants.

Similar findings were observed in POD, changes in the POD enzyme activity in the leaf were assessed at the full irrigation (1.0 ETc) and drought stress levels (0.75 and 0.50 ETc treatments). Grafting increased the amount of this enzyme in grafted cucumber plants compared to nongrafted plants (Fig. 6B). Nongrafted plants had lower POD enzyme activity but higher enzyme activity. The maximum POD enzyme activity was observed on the leaf of the grafted cucumber on Gurad2, followed by Guard 1, Just, Super green, and Colocynthoides rootstocks compared to nongrafted plants.

*The dynamic changes in* the *proline levels were detected in* the *non-grafted (scion) plants, and five rootstock-grafted plants* under normal and *drought stress* conditions (Fig. 6C). *Under drought conditions,, proline levels revealed the significant increases of five root stock plants* compared with control plants*., the* proline content in the *rootstock-grafted plants* increased by *52.17%, 47.62%, 50%, 45% and 31.25%* at 0.75 ETc and*,*46.34%, 37.14%,38.88%, 18.51% and 12% at 0.50 ETc for cucumber plants grafted on *Super green, Just, Guard 1, Guard 2 and C.L.Colocynthoides*, respectivaly.*No significant differences in proline levels were observed among* grafted and nongrafted *plants under control conditions* (1.0 ETc)0.3.4.5. *Leaf mineral content.*

The concentration of mineral elements (N, P, K, Ca, Mg, Fe, and Zn) was significantly affected by used rootstocks and water levels (Table 4). The grafted cucumber plants accumulated more minerals than nongrafted plants under normal and stressful conditions. The concentration of mineral content in cucumber leaves gradually increased with the increasing irrigation water level. The maximum leaf mineral concentrations were recorded in full irrigation conditions (1.0 ETc), followed by moderate (0.75 ETc), whereas the minimum values were observed in severe drought conditions (0.50 ETc).

**Table 4:** Effect of interaction of water regimes and rootstocks on leaf nutrient content. Different letters indicate significant differences between treatments (Duncan’s multiple range test at *p* < 0.05) and ± value indicates to stander error

Root stock	Treatment	N (%)	P (%)	K (%)	Mg (%)	Ca (%)	Fe (ppm)	Zn (ppm)
**Super green**	**1.00 ETc**	4.120 ± 0.11 a	0.47 ± 0.07 a	3.62 ± 0.21 ab	0.65 ± 0.021 a	2.51 ± 0.10 a	198.1 ± 9.1 a	86.27 ± 4.11 b
	**0.75 ETc**	3.590 ± 0.12 c	0.38 ± 0.009 c	3.02 ± 0.21 fg	0.48 ± 0.041 e	2.10 ± 0.10 bc	139.8 ± 2.1 bc	77.36 ± 2.09 f
	**0.50 ETc**	2.550 ± 0.91 gh	0.23 ± 0.002 ij	2.83 ± 0.44 j	0.28 ± 0.01 l l	1.90 ± 0.10 e–g	78.19 ± 8.1 fg	51.13 ± 5.10 n
**Just**	**1.00 ETc**	4.190 ± 0.71 a	0.41 ± 0.05 b	3.580 ± 0.51 c	0.63 ± 0.010 b	2.50 ± 0.10 a	200.1 ± 25.10 a	82.31 ± 1.10 c
	**0.75 ETc**	3.310 ± 0.21 d	0.35 ± 0.077 d	3.02 ± 0.21 fg	0.45 ± 0.019 f	2.11 ± 0.10 bc	135.9 ± 31.10 bc	75.49 ± 6.3 g
	**0.50 ETc**	2.630 ± 0.10 j	0.21 ± 0.097 kl	2.92 ± 0.11 i	0.25 ± 0.067 m	1.88 ± 0.10 fg	78.95 ± 4.1 fg	48.72 ± 1.15 o
**Guard 1**	**1.00 ETc**	4.150 ± 0.13 a	0.31 ± 0.065 e	3.61 ± 0.71 b	0.62 ± 0.077 bc	2.55 ± 0.10 a	204.3 ± 56.1 a	73.44 ± 7.10 h
	**0.75 ETc**	3.020 ± 0.81 e	0.25 ± 0.024 h	3.03 ± 0.56 f	0.42 ± 0.036 g	2.08 ± 0.10 b-d	130.2 ± 59.1b-d	58.23 ± 9.12 k
	**0.50 ETc**	2.530 ± 0.10 h	0.24 ± 0.080 hi	2.80 ± 0.24 k	0.25 ± 0.034 m	1.81 ± 0.10 g	101.4 ± 9.1 d-e	45.11 ± 2.16 p
**Guard 2**	**1.00 ETc**	4.210 ± 0.19 a	0.46 ± 0.091 a	3.63 ± 0.031 a	0.59 ± 0.056 d	2.53 ± 0.10 a	203.3 ± 34.1 a	88.86 ± 3.12 a
	**0.75 ETc**	3.250 ± 0.18 d	0.27 ± 0.02	3.01 ± 1.01 g	0.38 ± 0.087 i	2.03 ± 0.10 c-e	125.7 ± 88.1b-e	70.40 ± 5.10 i
	**0.50 ETc**	2.580 ± 0.20 gh	0.24 ± 0.071 hi	2.79 ± 0.91 k	0.23 ± 0.090 n	1.85 ± 0.10 fg	100.3 ± 13.11 d-e	41.09 ± 1.90 q
**C.L. Colocynthoides**	**1.00 ETc**	4.140 ± 0.88 a	0.42 ± 0.051 b	3.57 ± 0.31 c	0.61 ± 0.017 c	2.20 ± 0.10 b	191.2 ± 16.15 a	81.21 ± 3.89 d
	**0.75 ETc**	3.240 ± 0.15 d	0.29 ± 0.010 f	3.05 ± 0.71 e	0.40 ± 0.030 h	2.01 ± 0.10 c-e	120.4 ± 0.12b-e	62.83 ± 3.17 j
	**0.50 ETc**	2.570 ± 0.58 gh	0.22 ± 0.061 jk	2.94 ± 0.93 h	0.30 ± 0.044 k	1.80 ± 0.10 g	98.96 ± 20.12 ef	55.29 ± 4.10 m
**Luarens**	**1.00 ETc**	3.90 ± 0.21 ab	0.39 ± 0.021 c	3.11 ± 0.98 d	0.46 ± 0.056 f	2.15 ± 0.10 bc	147.1 ± 19.16 b	80.86 ± 4.51 e
	**0.75 ETc**	2.860 ± 0.40 f	0.21 ± 0.013 kl	2.91 ± 0.31 i	0.35 ± 0.077 j	1.95 ± 0.10 d-f	114.6 ± 11.13 c-e	56.23 ± 5.01 l
	**0.50 ETc**	2.010 ± 0.01 i	0.18 ± 0.041 m	2.74 ± 0.11 l	0.20 ± 0.094 o	1.24 ± 0.10 h	51.72 ± 8.15 h	37.08 ± 2.99 r

### Expression of Argonaut (*CsAGO1*) and RNA-dependent RNA polymerase (*CsRDR1*) genes in cucumber plants under drought stress conditions

**Fig. 7:**
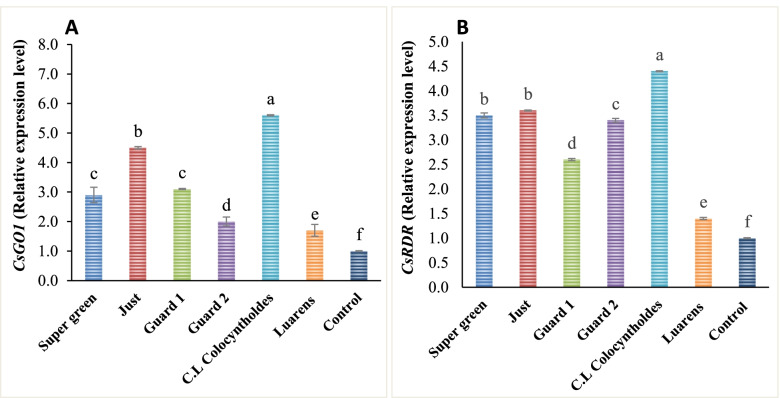
Relative expression level of *CsRDR1* and *CsGO1* genes of the used rootstocks. Significance of differences, *P* < 0.001 (**), between means was evaluated by Student’s t-test, ± SE (*n* = 4)

This investigation confirmed the molecular regulation of rootstock-grafting and non-drafting plants at the transcriptional level during drought stress. The transcript levels of the drought stress-responsiveness of Argonaut (*CsSAGO1*) and RNA-dependent polymerase (*CSsRDR1*) were performed by quantitative real-time PCR in rootstock-grafted plants and non-grafted (scion plant) during drought stress (0.75 ETc) and full irrigation conditions (control, 1.0 ETc) as presented in Fig. 7 A and B. The results showed that transcript levels of *CsAGO1* genes in non-grafted Laurens did not significantly change under full irrigation conditions (control*, 1.0 *ETc*)*, whereas *CsAGO1* gene transcript levels were increased in five rootstock-grafted studied types. This gene’s mRNA levels were higher in rootstock-grafted watermelon (*Citrullus* Lanatus var. Colocynthoides) than in non-grafted plants Laurens. As a result, *CsAGO1* transcript levels increased by 5.6 folds in Colocynthoides, 4.5 folds in Just, 3.1 folds in Guard 1, 2.8 folds in Super Green, and 2.0 times in Guard2 While the CSAGO1 transcript levels decreased to 1.7 fold in non-grafted Laurens (scion plant) under drought stress as presented in Fig. 7 A. Besides that, after 6 days of drought treatment (0.75ETc), the *CSRDR1* gene transcript levels rapidly improved in the five rootstock-grafted plants but downregulated in nongrafted plants. The transcript levels of the *CsRDR1* gene remained elevated after 6-day stress. A 4.4-fold in Colocynthoides rootstock-grafted plants, 3.6-fold in Just, 2.6-fold in Guard1, 3.5-fold in Super Green, and 3.4 fold in Guard 2 were observed and decreased in to 1.4 fold in non-grafted Laurens scion plant) under drought stress (Fig. 7 B).

These differences in expression analysis of the prospect genes under 0.75 ETc recommend that they play an important role in the molecular mechanism network during the water deficiency stress-response. In this study, *CsAGO1* and *CsDCLs* were found to be highly gene expressed in the leaves. These findings suggest that *CsAGO1* and *CsDCLs* play a role in the leaf development during vegetative and reproductive development. Differences in *CsAGO1* and *CsRDR1* gene expressions in rootstock under water-deficit stress treatment show that leaves are the plant’s most drought-sensitive organ.

The current study’s findings will encourage additional functional characterization of *CsAGO1* and *CsDCLs* genes in plant stress responses, potentially improving our understanding of the complex stress-response molecular network in rootstock plants. At the moment, *CsAGO1* and *CsDCLs* gene overexpression lead to a higher tolerance of water deficits during early plant development in rootstock-grafted cucumber plants. *CsAGO1* and *CsDCLs* genes, both are miRNAs, have emerged as critical candidates for managing plants’ responses to abiotic stress.

The findings show that the studied rootstock-grafted plants enhance drought tolerance, probably by influencing transcriptional regulation of stress-related regulatory and functional genes in an immediate and long-term manner. The mRNA transcript levels of two stress-responsive genes, Argonaut (*CsAGO1*) and RNA-dependent RNA polymerase (*CsRDR1*) were higher in rootstock-grafted than in nongrafted plants. Through their constitutive use as primary components of RNA-stimulated silencing complexes, which activate the RNA silencing mechanism, *CsAGO1* and *CsRDR1* genes play an important role in combating cellular dehydration.

### Correlation study

A correlation-based method using the Pearson coefficient was performed to detect positive and negative relationships among agro-physicochemical attributes of grafted cucumber plants treated with different irrigation water levels (Table 5). The Pearson’s correlation analysis showed the total yield of grafted cucumber plants was positively correlated with plant growth traits, including (LWC), the number of leaves, plant height, root dry matter shoots dry matter, RLA, and RSE. A similar correlation also was observed between total yield and physiological parameters, such as leaf nutrient content (N, P, K, Ca, Mg, Fe, and Zn), chlorophyll content, chlorophyll fluorescence, and GA3. Conversely, a negative correlation was noted between the yields of grafted cucumber plants and ABA and MDA.

**Table 5:**
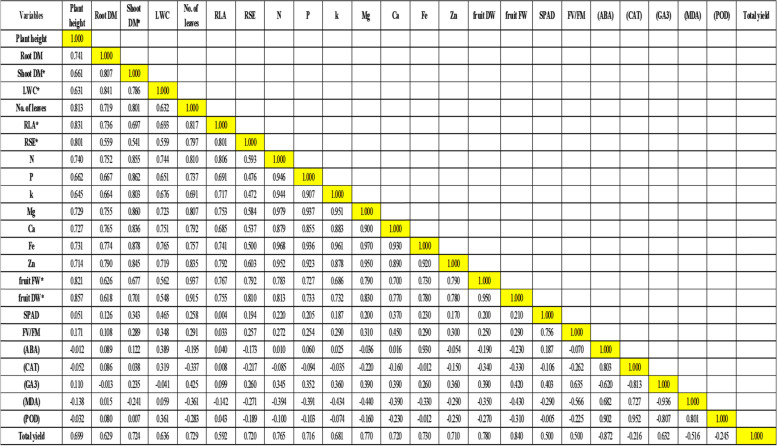
Pearson’s correlation analysis between the agro-physiological properties of grafted and non-grafted cucumber plants treated with different levels of irrigation water

These findings suggested that cucumber grafting on rootstocks of the Battle Guard1, Battle Guard 2, and Watermelon (Colocynthoides) were effective to improve the cucumber growth performance and induce tolerance against drought stress by modulating the physiological processes and mitigating oxidative stress in plant cells mainly under drought conditions.

## Discussion

Drought is the most critical environmental and multidimensional stress that causes a reduction in the agronomical, biochemical, and physiological aspects of cucumber plants [27, 29, 30]. Cucumber is the main vegetable and has a high sensitivity to water insufficiency stress that destructively influences plant growth, fruit quality, and yield production [31]. The cultivation of cucumber plants under water-deficit stress reduces their qualitative and quantitative characteristics, resulting in serious commercial problems for cucumber growers. The chlorophyll content of cucumber leaves is an important marker of environmental stress [32]. Decreased chlorophyll ratio under drought conditions has increased the pigment destruction and disrupted the antioxidant enzyme activities that are responsible for the synthesis of photosynthesis pigments [33]. Moreover, the negative influence of the plant height, stem numbers, and fresh and dry weight under drought might be associated with reduced plant hormones, plant development, LA extension, and insignificant radiation quantity blocked during decreased photosynthesis. Under drought, the photosynthesis instrument is disrupted due to lower electron transportation through PS II and/or structural damage to PS II and the light-harvesting complex [34].

Various mechanisms can control drought stress, including grafting and genetic breeding. Lately, the grafting system has been used to maintain drought stress in numerous plant types [35, 36]. To our knowledge, the current study is the first to explore and choose five rootstock plants tolerant to water deficits, which may cause drought tolerance in cucumbers by activating the stress-responsive gene expression and antioxidant enzyme system [4]. In this regard, the application of the grafting mechanism noticeably decreased the destructive effects of drought stress and improved the plant development, LA, LWC, leaf numbers, plant height, the leaf chlorophyll, and shoot and root dry matter [37].

This study revealed that improved growth parameters were associated with a higher irrigation rate that may be documented to the suitable balance of moisture range produced by plant tissues. This moisture equilibrium constructs appropriate conditions for the uptake of nutrients, photosynthesis, and metabolite growth. In this study, grafting with novel water-deficit-tolerant rootstocks could enhance drought tolerance and yield production by improving antioxidant enzyme activities, phytohormones, and stress-responsive gene expression in cucumbers during two seasons.

Results of physiological parameters in five rootstock-grafted plants have increased the growth under water deficiency stress. The effects of grafting methods on cucumber growth parameters demonstrated a significant interaction between the studied scion and rootstock plants. These results proved that five rootstock genotypes played an essential role in drought tolerance. This positive outcome may be frequently attributable to the novel root systems of studied genotypes, which are recognized as rootstocks that are noticeably longer, stronger, and tolerant to drought stress. In this regard, these five novel rootstocks absorbed water and nutrients more effectively than nongrafted genotypes. Plant water relations have a critical role in stimulating stress-responsive gene expression and antioxidant defense mechanisms during drought. Moreover, grafting led to increased plant growth and productivity based on the strongly selected rootstocks. Furthermore, the present study is consistent with the outcomes of [37–39], who revealed that rootstock-grafted plants were stronger and larger than nongrafted genotypes.

In this study, our results from five rootstock grafting plants showed a higher chlorophyll ratio than the nongrafted. These results proved that rootstock grafts reduced the inhibition of photosynthesis induced by water-deficit stress. Five novel rootstock grafting studies revealed that plants preserved the biological function of PSII while protecting their high photosynthetic capability under water deficiency stress. These outcomes were validated by lowering transpiration, and the rootstock might improve drought resistance and water content in response to water scarcity. Reduced nongrafted plants’ photosynthetic activity showed their lack of ability to adjust to soil water deficiency situations. Furthermore, the photochemical efficiency of the nongrafted cucumber leaves was decreased due to light energy absorption. A decrease in chlorophyll concentration in nongrafted plants is a drought-response mechanism to reduce light absorption by chloroplasts [40]. Drought-induced chlorophyll depletion is thought to be a unique oxidative stress signal caused by pigment photooxidation [41].

In this regard, under water scarcity conditions, the chlorophyll content of grafted rootstock tomato genotypes is higher [42]. In muskmelon plants, grafted rootstocks boost the photosynthetic system and sugar transfer [6]. The results confirmed that the grafting technique caused numerous changes in the chemical composition and mineral numbers in the cucumber plant tissues, following the same pattern. Our results demonstrated that the control treatment (1.0 ETc) increased the plant growth, followed by moderate drought management (0.75 ETc) and finally severe drought (0.50 ETc).

The current findings are consistent with those of [42, 43], who found that cucumber plants were cultivated under the full irrigation treatment (1.0 ETc), followed by a drought treatment (0.8 ETc), which exhibited significantly greater vegetative growth than plants cultivated under a lower irrigation condition. When five rootstock plants were correlated with the full irrigation condition and (0.75 ETc) drought treatment, the most elevated growth, yield, and production traits were increased. Our findings demonstrated a link between improved plant growth and increased irrigation conditions, resulting in a suitable balance of moisture concentration produced by plant tissues. This moisture equilibrium creates favorable conditions for photosynthesis capacity, nutrient uptake, and metabolite translocation, plants grow at a rapid rate as a result. These findings suggest that the grafting method alleviates the adverse impacts of water-deficit stress.

Plants tend to create excessive reactive oxygen signals in response to drought stress. By detoxifying O2 radicals and creating H2O2, antioxidant enzymes like POD and catalase help prevent cellular damage. In this study, antioxidant enzymes, POD and catalase, increased in rootstock-grafted examined plants but decreased in nongrafted genotypes under water deficiency stress conditions. This shows that catalase detoxification is completed efficiently in grafted plants, allowing them to detoxify larger H2O2 levels and hence protect plants from reactive oxygen signal toxicity during water deficiency stress [44, 45]. In this regard, the five local varieties of rootstock-grafted plants have a more effective tolerance to drought, as demonstrated by the development of an antioxidant system, indicating high growth performance. Furthermore, during drought stress conditions, lipid peroxidation in another pathway was reduced in five local varieties of rootstock-grafted plants to enhance tolerance to water-deficit conditions. Jianjun et al. [6] revealed that the rootstock-grafted plants showed much higher SOD, CAT enzyme activities, and proline content than non-grafted and self-grafted plants in drought stress conditions in tobacco plants. In this way, grafted plants achieve greater tolerance to drought stress, apparently by developing a better antioxidant system and non-anatioxidant, which in turn leads to better growth performance.

The current study found that rootstock-grafted genotypes may improve water deficiency tolerance by activating stress-response genes during drought. These results show that high ABA levels in rootstock-grafted plants exhibit enhanced drought tolerance, possibly due to long-distance signaling ABA synthesis, transportation, and regulation of metabolic pathways in cucumber cellular and whole-plant stages. The findings revealed that the grafting process increased the levels of the most important hormones, such as GA3 and that increased GA3 levels in rootstock plants control several vital physiochemical developments in plants, i.e., secondary growth and xylem fiber extension in response to drought. Furthermore, the two regulatory genes were elevated at higher levels in rootstock-grafted genotypes than in nongrafted genotypes, implying that ABA and phytohormone (GA3) signaling may be boosted in cucumber rootstock grafting-mediated water shortage responses.

Further studies on five cucumber rootstocks revealed that stress-responsive genes were activated, resulting in improved water-deficit stress tolerance in cucumber plants. Based on these findings, drought stress-responsiveness of Argonaut (*CsAGO1*) and RNA-dependent RNA polymerase (*CSRDR1*) genes were found to be higher in five rootstock cucumber plants than in nondrafted plants. These genes (*CSAGO1* and *CSRDR1*) are the major components of a display of siRNAs used to enhance plant development and tolerance to drought [44, 45]. In this respect, Gan et al. [46] revealed that the gene expression levels of AGO and RDR in cucumber were expressed under drought stress treatments, providing insights into stress tolerance mechanisms and other physiological processes.

Yang and Zhong [47] reported that upregulation of the *OsAGOs* gene results in a response to light and dark treatments. Shao and Lu [48] showed that the SmRDRs gene has the most enhanced expression in roots and the lowest expression levels in flowers and leaves in response to scarcity stress.

The rootstock-grafted plants that expressed oxidase 1 (ACO1) silencer gene, which controls developmental stress responses and chromatin structure by protecting membrane integrity and efficiently pressing water, which both are necessary processes for plants to protect plant growth under drought. Gan et al. [46] found that high expression of Dicer-like (DCL), argonaut (AGO), and RNA-dependent RNA polymerase (RDR) genes resulted in the RNA silencing modulation to promote cucumber growth and response to abiotic stress. Grafting could also cause rootstock-source signal transmission based on this evidence. Further studies indicate that rootstock-induced water-deficit stress responses in cucumber plants can mimic several signals. These signals are important in clarifying the regulatory and molecular mechanisms of grafting and in hastening the potential grafting application in improving drought tolerance in cucumber plants.

## Conclusions

According to current agronomical, physiological, and molecular characteristics, the choice of a robust rootstock is the most important factor in determining cucumber water deficit tolerance. The present study demonstrated that grafting using local variants of unique rootstock genotypes can help cucumber plants under drought stress improve their phenotypic, physiological, and water usage efficiency-related properties.

Laurens was grafted into rootstocks such as Super Green, Just, Battle Guard 1, Battle Guard 2, and watermelon. Grafting cucumber plants on rootstocks enhanced the morphological traits of cucumber plant under normal and stressful conditions. Moreover, to induce the plant's tolerance to drought through increasing the expression of defense related genes and increasing accumulation of GA3 and activity of antioxidant enzymes (CAT and POD), chlorophyll content, Chlorophyll fluorescence, efficiency of photosystem II (PSII) and decreasing the electrolyte leakage, ABA, and MDA, especially under drought conditions (0.75ETc and 0.50ETc).

Furthermore, crop output can be increased by combining five strong rootstocks with the vigorous commercial genotype Laurens as a scion. In conclusion, the current study indicated several advantages, including increased commercial productivity while conserving water, which is the primary goal of greenhouse cucumber growers.

## Data Availability

Not applicable.
